# Construction, Expression, and Characterization of a Recombinant Immunotoxin Targeting EpCAM

**DOI:** 10.1155/2015/460264

**Published:** 2015-04-16

**Authors:** Minghua Lv, Feng Qiu, Tingting Li, Yuanjie Sun, Chunmei Zhang, Ping Zhu, Xiaokun Qi, Jun Wan, Kun Yang, Kui Zhang

**Affiliations:** ^1^Department of Clinical Immunology, Xijing Hospital, Fourth Military Medical University, Xi'an 710032, China; ^2^Department of Neurology, Chinese Navy General Hospital, Beijing 100048, China; ^3^Department of Geriatric Gastroenterology, Chinese People's Liberation Army General Hospital, Beijing 100853, China; ^4^Department of Immunology, Fourth Military Medical University, Xi'an 710032, China

## Abstract

Epithelial cell adhesion molecule (EpCAM) is a type I transmembrane glycoprotein overexpressed in human epithelioma but with relatively low expression in normal epithelial tissues. To exploit this differential expression pattern for targeted cancer therapy, an EpCAM-targeted immunotoxin was developed and its antitumor activity was investigated *in vitro*. An immunotoxin (scFv2A9-PE or APE) was constructed by genetically fusing a truncated form (PE38KDEL) of *Pseudomonas aeruginosa* exotoxin with an anti-EpCAM single-chain variable fragment (scFv). ELISA and flow cytometry were performed to verify immunotoxin (scFv2A9-PE or APE) antigen-binding activity with EpCAM. Cytotoxicity was measured by MTT assay. Confocal microscopy was used to observe its cellular localization. The results of ELISA and flow cytometry revealed that the immunotoxin efficiently recognized recombinant and natural EpCAM. Its antigen-binding activity was relatively lower than 2A9. MTT assay confirmed potent reduction in EpCAM-positive HHCC (human hepatocellular carcinoma) cell viability (IC_50_ 50 pM). Immunofluorescence revealed that the immunotoxin localized to endoplasmic reticulum 24 h later. In conclusion, we described the development of an EpCAM-targeted immunotoxin with potent activity against tumor cells, which may lay the foundation for future development of therapeutic antibody for the treatment of EpCAM-positive tumors.

## 1. Introduction

The most ideal outcomes for tumor targeted therapy are improved patient survival and minimal adverse effects on normal tissues. Drugs that can specifically home to a cancer cell based on a surface receptor have helped to address that goal with the advent of monoclonal antibody therapy [1]. Rituximab, directed against the CD20 antigen found on the surface of normal and malignant B cells, is the first monoclonal antibody approved by the US Food and Drug Administration for the treatment of B-cell non-Hodgkin's lymphoma [2]. Monoclonal antibodies have been developed and have been impressive, but they are limited by immunogenicity [3], thus, leading to the development of single-chain variable fragment (scFv) antibodies.

Researchers have designed and produced many scFvs since the 1980s. Recombinant scFvs are promising because they can target an effector molecule or a cell to a disease-related target structure [4, 5]. Immunotoxin, as one type of immunoconjugate, can be produced by genetically fusing scFv with toxin and this molecule can recognize target cells by scFv and kill them via its toxin. Many immunotoxins have undergone or are currently undergoing study in humans for leukemia treatment [6, 7]. Denileukin diftitox (Ontak) has been approved by the FDA for the treatment of cutaneous T-cell lymphoma in adults [8]. Thus, immunotoxins are promising therapeutics for targeted cancer therapy.

Epithelial cell adhesion molecule (EpCAM), also known as CD326, is a type I membrane glycoprotein of approximately 40 kDa. It participates in many biological processes, such as cell adhesion, proliferation, and differentiation [9]. EpCAM is frequently highly expressed on most solid tumors, including carcinomas of the breast, ovarian, lung, colon, and pancreatic cancer and in squamous cell carcinoma of the head and neck, suggesting its potential as a therapeutic target [10, 11].

EpCAM-targeted antibody therapy has been studied frequently since the 1980s. MAb17-1A, a low affinity monoclonal antibody against EpCAM, is successfully used in Germany for breast and colon carcinoma therapy [12, 13] and CD3/17-1A, a bispecific scFv, is demonstrated to have cytotoxicity to EpCAM-positive tumor cells* in vitro* [14]. Finally, catumaxomab, a trifunctional anti-EpCAM/CD3 monoclonal antibody, has been approved in the European Union for the treatment of EpCAM-positive tumors in patients with malignant ascites [15]. Due to limited applications and adverse effects of these antibodies, researchers wish to exploit more effective and EpCAM antibodies with greater potential to treat carcinomas. In the past 20 years, fully humanized and bispecific scFv fusion proteins have been studied in preclinical and clinical trials [16, 17] and EpCAM targeted immunotoxins have been confirmed to have antitumor activity* in vitro* [18]. Simon made modification to an EpCAM-targeting fusion toxin by facile click PEGylation to increase its antitumor efficacy* in vitro* and* in vivo* [19]. All these investigations have increased the promise of EpCAM as a target for cancer therapy.

We prepared seven EpCAM monoclonal antibodies, FMU-EpCAM-2A9, FMU-EpCAM-2D7, FMU-EpCAM-4B11, FMU-EpCAM-4F11, FMU-EpCAM-4E4, FMU-EpCAM-4A11, and FMU-EpCAM-4F6. FMU-EpCAM-2A9 and FMU-EPCAM-2D7 are also named FMU-Ep1 and FMU-Ep3, respectively. In previous work, we reported that some of these antibodies (FMU-Ep1 and FMU-Ep3) can be used for immunohistochemical staining to identify normal and malignant colon tissue [20]. However, whether these are effective anticancer agents is uncertain. Thus, we report the construction, expression, and characterization of an immunotoxin, comprised of a single-chain variable fragment (scFv) of FMU-EpCAM-2A9 and a truncated form (PE38KDEL) of* Pseudomonas aeruginosa* exotoxin. The recombinant immunotoxin was successfully cloned and expressed and its antigen-binding ability and cytotoxicity were measured. This recombinant immunotoxin potently inhibited HHCC cell lines, which lays the foundation for further development of this agent as a possible cancer chemotherapeutic.

## 2. Materials and Methods

### 2.1. Materials

The recombinant plasmid PGEX-4T3-EpCAM and the monoclonal antibodies of EpCAM (FMU-EpCAM-2A9 and FMU-EpCAM-2D7) are all prepared in our lab. Fetal bovine serum and mRNA isolation kit are purchased from Gibco. FITC conjugated goat anti-mouse IgG is bought from Biolegend. Mouse anti-GST antibody, protein ultrafiltration centrifugal tube, and PVDF membrane are from Millipore. The primers used were synthesized by Shanghai Sangon Biotech Company. The sequences of the primers were listed in Table 1.

### 2.2. Organism

*Escherichia coli* DH5*α* and BL21 were used for cloning of the pMD-T18-2A9-V_H_ (or -V_L_) plasmid and pGEX-4T1-scFv plasmid, respectively.* E. coli* M15 was used to express the extracellular domain of EpCAM (pQE30-EpCAM).

### 2.3. Cell Lines and Cultures

The hepatocellular carcinoma cell lines (HHCC and SMMC-7721), breast cancer cell line (SKBR3), and colon cancer cells (Colo205 and SW480) are all from ATCC. They were grown in RPMI 1640 medium supplemented with 10% fetal bovine serum (FBS) and 1% penicillin-streptomycin (100 units/mL penicillin and 100 *μ*g/mL streptomycin) at 37°C and 5% CO_2_ in a humidified incubator.

### 2.4. Cloning Light and Heavy Chain Variable Region of FMU-EpCAM-2A9 (FMU-Ep1)

Hybridoma cells were cultured in RPMI1640 medium supplemented with 10% fetal bovine serum. Cells were collected in the logarithmic phase and total RNA was extracted with Trizol according to the manufacturer's instructions. The sequences encoding the light and heavy chain variable regions (V_L_ and V_H_) of 2A9 were amplified by RT-PCR. The V_H_ sequence was amplified using primers A and B, while the V_L_ sequence was amplified with primers C and D. After purification, PCR products were cloned into a pMD-T18 vector and transformed into* E. coli* DH5*α*. The positive colony (pMD-T18-2A9-V_H_ or pMD-T18-2A9-V_L_) was identified by colony PCR and restriction enzyme analysis.

### 2.5. Construction of the pGEX-4T1-scFv2A9-PE Expression Vector

The sequence encoding the 2A9-V_H_ and 2A9-V_L_ was amplified by PCR from plasmids pMD-T18-2A9-V_H_ and pMD-T18-2A9-V_L_, respectively, and inserted into the pGEX-4T1 expression vector in two steps. A special linker was added to the N terminal of V_L_ by forward primer. The peptide sequence of the linker was GGGGSGGGGSGGGGS. The 2A9-V_H_ sequence was amplified by primers E and F, whereas the 2A9-V_L_ sequence was amplified using primers G and H. After gel purification, the amplified V_H_ products were digested, purified, and ligated between the BamH I and Sal I sites of plasmid vector pGEX-4T1. After identification of the pGEX-4T1-V_H_ plasmid, the amplified V_L_ products were ligated between the Sal I and EcoR I sites of plasmid vector pGEX-4T1-V_H_. The pGEX-4T1-V_H_-V_L_ (pGEX-4T1-scFv2A9) vector was confirmed by restriction enzyme digestion and DNA sequencing. The sequence encoding a truncated form of* Pseudomonas* exotoxin (PE38KDEL) was amplified by PCR, and the template was kindly supplied by Professor Boquan Jin of the Fourth Military Medical University and cloned as an ~1,200 bp EcoR I-Xhol I fragment downstream of the scFv2A9 sequence present in the pGEX-4T1-based scFv2A9 expression vector. The primers used were I and J. The expression vector pGEX-4T1-scFv2A9-PE was identified by restriction enzyme digestion and DNA sequencing.

### 2.6. Protein Expression and Purification of the Immunotoxin against EpCAM

The pGEX-4T1-scFv2A9-PE plasmid was expressed in BL21* E. coli* cells. Bacterial cultures were incubated at 37°C in LB growth medium containing 100 ng/mL ampicillin and grown until an early log phase (A600 nm = 0.6–0.8). Protein expression was induced for 7 h at 30°C by the addition of IPTG (final concentration 500 nM). Bacteria were harvested by centrifugation at 12,000 rpm for 20 min at 4°C. For purification, the pellet obtained from a 100 mL culture was resuspended in 10 mL 0.15 M PBS and pulse-sonicated for 30 × 1 min (1 s working and 1 s resting for a 1 min pulse and then cooled on ice for 1 min). The soluble and insoluble fractions were separated by centrifugation at 12,000 rpm for 20 min at 4°C. Then the soluble fraction was purified by using the Glutathione Resin GST Fusion Protein Purification Kit according to the manufacturer's instructions (Genscript cat. number L00206). The purified immunotoxin was labeled by biotin according to the manufacturer's instruction (Roche, Biotin Protein Labeling Kit).

### 2.7. Western Blotting Detection of the Immunotoxin against EpCAM

Purified protein samples were analyzed by electrophoresis on 10% SDS-PAGE under denaturing conditions and transferred to PVDF membrane. Western blot analysis was conducted using a mouse anti-GST antibody (the scFv was coexpressed with a GST tag) as the primary antibody (Millipore, 1 : 3000) and a horseradish peroxidase- (HRP-) labeled rabbit anti-mouse IgG as the secondary antibody, in accordance with the manufacturer's protocols.

### 2.8. Construction of the pQE30-EpCAM Plasmid

The sequence encoding the extracellular domain of EpCAM was amplified by PCR from plasmid pGEX-4T-3-EpCAM and cloned as a 750 bp Kpn I - Hind III fragment to the plasmid pQE30. The primers used were K and L.

### 2.9. Prokaryotic Expression of pQE30-EpCAM

The pQE30-EpCAM plasmid was used to express the extracellular domain of EpCAM in M15* E. coli*. Cells were grown at 37°C in a shaking incubator (220 rpm) until the culture reached an OD_600_ of 0.6–0.8. Protein expression was induced for 7 h at 30°C by adding IPTG (Sigma) at a final concentration of 500 nM. The harvested pellet was ultrasonicated and analyzed via SDS-PAGE Coomassie Blue staining and Western blot. The protein from the supernatant was purified with a Nickel-affinity chromatography column, in accordance with the manufacturer's instruction (GE).

### 2.10. ELISA Detection of the Binding Ability of the Immunotoxin to EpCAM

The binding ability of the immunotoxin to EpCAM was detected by ELISA. Briefly, a 96-well plate was coated with 100 *μ*L 5 *μ*g/mL EpCAM-HIS recombinant protein overnight at 4°C in PBS. After incubation and washing, 100 *μ*L 2A9 or immunotoxin was added to the wells at different concentrations (20, 2, 0.2, 0.02, and 0.002 *μ*g/mL) and incubated for 1 h at 37°C. A mouse anti-GST primary antibody (1 : 500) and a HRP-labeled rabbit anti-mouse secondary antibody (1 : 2,500) were used to detect 2A9 or immunotoxin; the tetramethylbenzidine (TMB) was used to develop the ELISA results. All experiments were repeated three times.

### 2.11. Flow Cytometry Analysis

Colo205 and HHCC cells at 5 × 10^6^ cells/mL were incubated with biotin labeled immunotoxin (20 *μ*g/mL) or antibodies against EpCAM (20 *μ*g/mL) for 40 minutes at 4°C. The cells were washed with PBS and then incubated with the FITC labeled avidin or antibodies for 30 minutes at 4°C. The fluorescence was examined by flow cytometry analysis using a FACScan flow cytometer (BD).

### 2.12. *In Vitro* Cytotoxicity Assay

Cytotoxic activity of the immunotoxin was measured with a standard MTT assay. Briefly, 4,000 HHCC cells were seeded in 96-well microplates in a total volume of 200 *μ*L of culture medium/well. Immunotoxin (2–32.5 M) was added and cells were incubated for 72 h under standard cell culture conditions. Then, 20 *μ*L of 5 mg/mL MTT solution was added to each well, and plates were incubated for 4 h at 37°C. Cell lysis and formazan solubilization were achieved by the addition of 150 *μ*L DMSO, and released formazan crystals were allowed to dissolve 10 min at 37°C. Absorption was quantified at 490 nm using a microplate reader. All experiments were measured in triplicate.

### 2.13. Immunofluorescence

Intracellular localization of the immunotoxin was observed by laser scanning confocal microscopy. Briefly, the HHCC cells were seeded in 24-well plates. After incubating for 8 h, biotin-labeled immunotoxin was added to the wells and incubated for different times. To localize the immunotoxin, cells were washed and fixed with 4% paraformaldehyde, and after blocking, cells were stained with primary antibody of the specific endoplasmic reticulum protein CRT (prepared in our laboratory). Fluorescence was measured by adding PE conjugated goat anti-mouse IgG and FITC-labeled avidin. Cell nuclei were stained with DAPI. After mounting, results were observed with laser scanning confocal microscopy.

### 2.14. Statistical Analysis

Differences between groups were determined using an unpaired two-tailed Student's *t*-test. Data analyses were performed using GraphPad Prism version 5.0 (GraphPad Software, San Diego, CA). For all tests, a *P* value less than 0.05 was considered significant.

## 3. Results

### 3.1. Screening Parental Antibody against EpCAM

**Figure 1:**
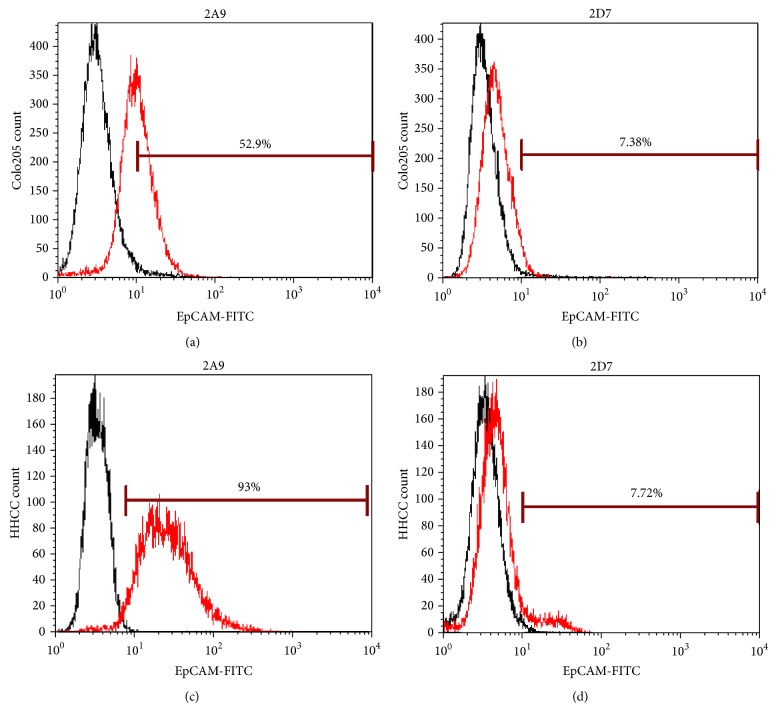
Binding ability of 2A9 and 2D7 to EpCAM was detected on Colo205 and HHCC cells by flow cytometry. Cells were incubated with biotin labeled EpCAM antibody (2A9 or 2D7, red histograms) or isotype control (black histograms) antibody (20 *μ*g/mL) for 40 min at 4°C. Then cells were washed and stained with FITC-labeled secondary antibody and analyzed by flow cytometry.

To screen the parental antibody of scFv, we compared the binding activity of two EpCAM mAbs (FMU-2A9 and FMU-2D7) with EpCAM on the surface of EpCAM-positive cells (Colo205 and HHCC) and EpCAM-negative cells (Sw480) by flow cytometry. Sw480 cells were similar to isotype control antibody (data not shown). The results of Colo205 and HHCC are depicted in Figure 1. 2A9 had relatively higher binding ability than 2D7 with the natural EpCAM molecule on these two cell surfaces. Thus, we selected 2A9 as the parental antibody to construct the scFv.

### 3.2. Construction, Expression, and Purification of the Immunotoxin

**Figure 2:**
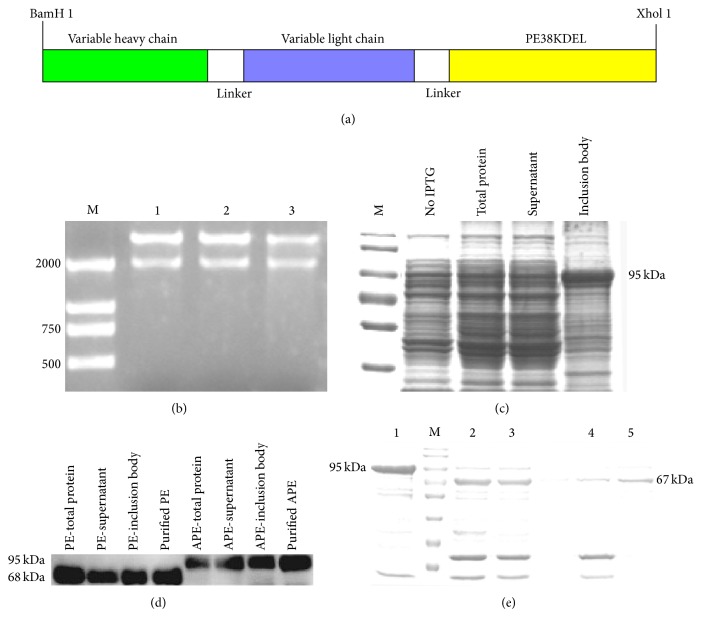
Construction, expression, and purification of immunotoxin. (a) Schematic representation of immunotoxin. (b) Restriction enzyme analysis of the expression plasmid (pGEX-4T1-scFv2A9-PE). Plasmids (lanes: 1–3) were digested with BamH-I and Xhol-I, and the 2,000 bp fragment was the recombinant immunotoxin. Lane: (M) LD2000 DNA ladder. (c) SDS-PAGE of recombinant protein. Protein from noninduced cells, IPTG induced cells, and supernatant and inclusion bodies were separated on 10% agarose gel and stained with Coomassie Brilliant Blue. Lane: (M) protein marker. (d) The recombinant protein was tested via Western blot. APE represents the immunotoxin; PE represents the empty control (pGEX-4T1-PE). (e) The purified recombinant protein was digested with thrombin. After removing the GST tag and thrombin, the immunotoxin was confirmed to be 67 kDa. Lanes: (1) purified APE protein; (M) protein marker; (2) APE after digestion with thrombin; (3) APE after digestion without thrombin; (4) APE without thrombin and GST protein; and (5) purified APE without the GST tag.

**Figure 3:**
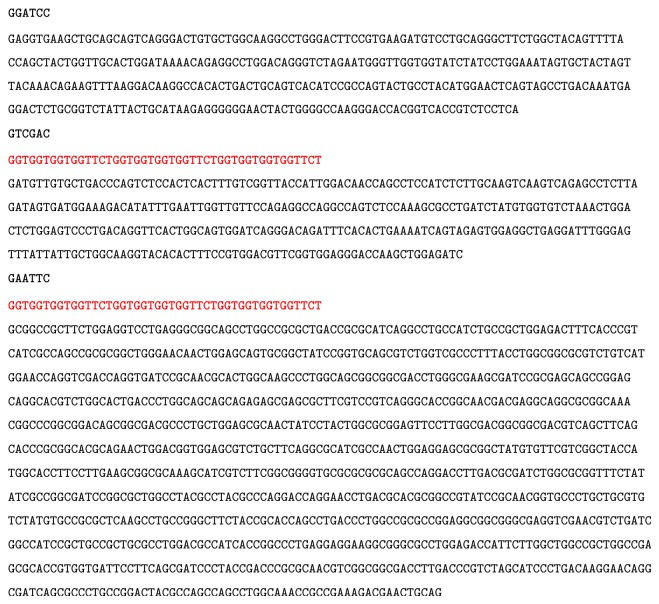
Sequence of immunotoxin. Black bold basic group represents restriction enzyme recognition site; red bold basic group represents the linker sequence.

After three rounds of PCR, the plasmid pGEX4T1-scFv2A9-PE was constructed. Three separate products generated an ~2,000 bp immunotoxin (Figures 2(a) and 2(b)). The sequence of the immunotoxin (APE) was further confirmed by DNA sequencing (Figure 3). APE was expressed in BL21* E. coli*, after sonication and purification, and samples were analyzed. SDS-PAGE and Western blot results (Figures 2(c) and 2(d)) indicated that the expressed GST-immunotoxin was ~95 kDa, which was consistent with the predicted molecule weight. Immunotoxin mainly interacted with inclusion bodies. To analyze immunotoxin activity, the GST tag was cut with thrombin and removed by purification, and the true molecule mass of the immunotoxin was 67 kDa (Figure 2(e)).

### 3.3. Construction, Expression, and Purification of the Extracellular Domain of EpCAM

**Figure 4:**
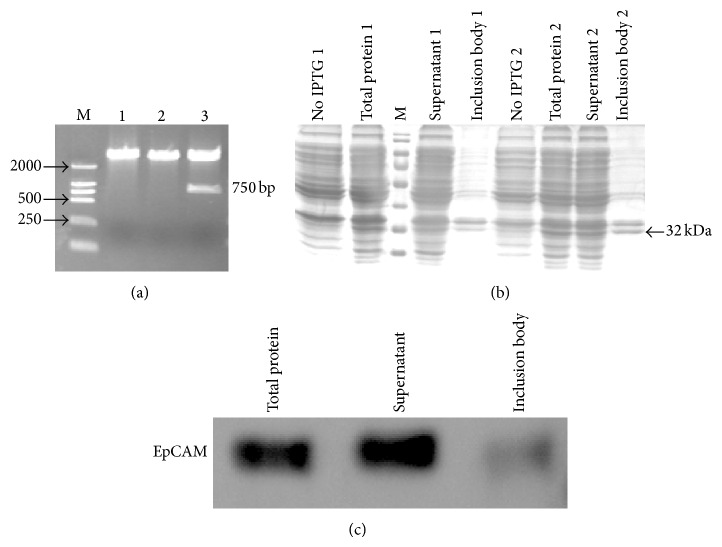
Restriction enzyme analysis, SDS-PAGE, and Western blot of recombinant EpCAM expressed in M15* E. coli* cells. (a) The plasmid pQE30-EpCAM was digested with Kpn I and Hind III. Lanes: (M) LD2000 DNA marker; (1–3) digestion products 1 and 2 were negative colonies; 3 was the positive colony. (b) Proteins were separated by 10% SDS-PAGE and visualized by Coomassie Brilliant Blue R250 staining. Two colonies were induced to express protein. (c) Purified HIS-EpCAM was identified by Western blot.

To analyze the binding ability of the immunotoxin, we constructed and expressed the recombinant protein HIS-EpCAM. The plasmid pQE30-EpCAM was identified by restriction enzyme analysis (Figure 4(a)) and DNA sequencing. The expression of HIS-EpCAM was induced with 500 nM IPTG at 30°C for 7 h. After sonication and centrifugation of the bacteria, total protein, supernatants, and inclusion bodies were separated with 10% SDS-PAGE under reducing conditions. As shown in Figure 4(b), the expression strain produced the recombinant protein in both soluble and inclusion bodies form. Western blot (Figure 4(c)) confirmed a novel band at the predicted molecular weight of 32 kDa. After purification and identification, proteins were stored at −80°C for ELISA.

### 3.4. Binding Ability of Immunotoxin to EpCAM

**Figure 5:**
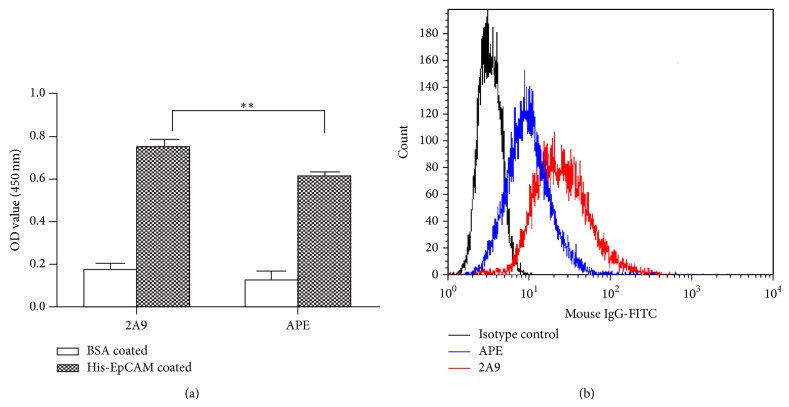
Binding ability of immunotoxin to recombinant His-EpCAM and natural EpCAM. (a) Recombinant HIS-EpCAM protein was coated on 96-well plates (5 *μ*g/mL). The binding of the immunotoxin to His-EpCAM was detected by ELISA. The *P* value was analyzed by an unpaired Student's *t*-test. ^∗∗^ *P* value was less than 0.05. (b) HHCC cells were collected and stained with primary antibody (biotin labeled immunotoxin, biotin labeled 2A9, or biotin labeled isotype control antibody) at a concentration of 5 *μ*g/mL and FITC labeled avidin (1 : 50) as the secondary antibody. Fluorescence was detected by BD FACS Calibur. The mean fluorescent intensity was measured by FlowJo software.

Immunotoxin was successfully prepared, and the binding ability of the immunotoxin to EpCAM was tested by flow cytometry and ELISA. Briefly, prepared His-EpCAM or BSA was coated on 96-well plates, and the immunotoxin or 2A9 was added to detect recognition to EpCAM. Data showed (Figure 5(a)) that 2A9 had relatively higher binding ability with recombinant His-EpCAM (2 *μ*g/mL) (unpaired *t*-test, *P* = 0.0071). In fact, the binding activity of APE with His-EpCAM was weaker than 2A9 at other concentrations used (20, 0.2, 0.02, and 0.002 *μ*g/mL, data not shown). Flow cytometry analysis (Figure 5(b)) demonstrated that the immunotoxin could efficiently recognize the EpCAM molecule on HHCC cells, although the binding ability was lower than 2A9. The mean fluorescent intensity of 2A9 was 25.02 ± 3.23, while the immunotoxin intensity was 10.03 ± 3.07.

### 3.5. Cytotoxicity Assay of Immunotoxin

**Figure 6:**
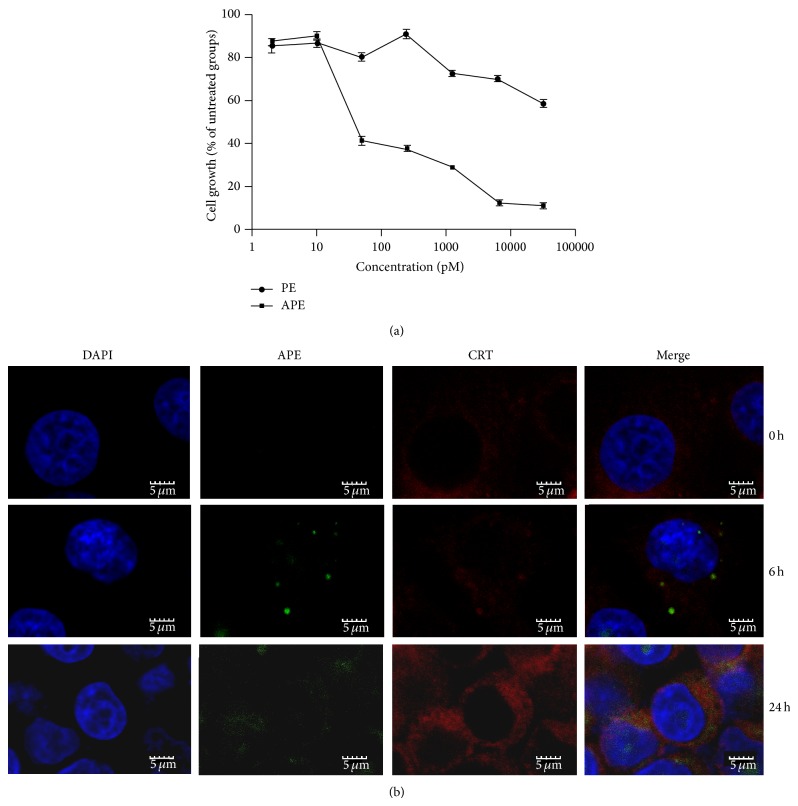
Cytotoxicity and localization of the immunotoxin. (a) HHCC cells were incubated with different concentrations (0.002, 0.01, 0.05, 0.25, 1.25, 6.5, and 32.5 nM) of the immunotoxin for 72 h, and cell growth was measured by MTT assay. (b) The localization of the immunotoxin was observed by laser scanning confocal microscopy at different incubation times (0, 6, and 24 h). DAPI indicates the cell nucleus; APE represents the immunotoxin; CRT is the calprotectin. All data are expressed as means ± standard deviations (SD).

The immunotoxin could bind to EpCAM in carcinoma cells, but whether it was cytotoxic to cancer cells requires more study. Cytotoxicity of the immunotoxin to EpCAM-positive (HHCC, Colo205, SMMC-7721, and SKBr3) and EpCAM-negative (Sw480) cells was tested by MTT assay. Data show (Figure 6(a)) that APE and PE both inhibited HHCC cell viability and had no other effect on other cells' survival (data not shown). The IC_50_ of the immunotoxin to HHCC cells was 50 pM, and the control toxin exceeded 5,000 pM. This indicates that the immunotoxin can efficiently recognize certain cancer cells and has anticancer activity.

The mechanism of cytotoxicity was studied by immunofluorescence. After staining, the localization of the immunotoxin was observed by laser scanning confocal microscopy. Data show that (Figure 6(b)) after 6 h of incubation, the immunotoxin internalized to the HHCC cells and colocalized with CRT protein in the endoplasmic reticulum where it diffused uniformly in the endoplasmic reticulum after 24 h of incubation.

## 4. Discussion

The challenging problem in cancer therapy is drug resistance and relapse. Thus, the development of novel drugs that target the specific antigen of carcinomas is greatly needed. EpCAM was reported to participate in the development and progression of diverse carcinomas as well as serve as a marker of prognosis [21], which triggered the study of EpCAM-target immunotherapy. EpCAM-specific antibodies were designed and used to treat many cancers* in vitro* and* in vivo* [22, 23]. Several EpCAM-target antibodies have been used in the clinic to treat malignant ascites and squamous cell carcinomas of the head and neck [24, 25], as well. These results suggest that EpCAM-targeted immunotoxin might be used to treat cancers.

In the current study, we designed, produced, and characterized a recombinant immunotoxin APE, comprised of an EpCAM scFv and PE38KDEL. We prepared seven EpCAM monoclonal antibodies, and they were used to identify CD326 at the 8th International Conference on HLDA (Human Leucocyte Differentiation Antigens) [26]. The sequences encoding the light and heavy chain variable regions of four antibodies have been cloned and homology comparison and analysis of the nucleoside sequences of the four variable regions were performed using GenBank + EMBL + DDBJ + PDB databases. The DNASIS program was used to analyze the nucleotide sequence and deduce the amino acid sequence, which was “blasted” in nonredundant GenBank CDS translations + PDB + SwissProt + PIR + PRF protein databases. IMGT/V-QUEST was used to analyze the structure of variable region and determine the CDR region of the antibodies. Patents have been sought for these four sequences of variable regions (antibodies were FMU-EpCAM-2A9, FMU-EpCAM-2D7, FMU-EpCAM-4E4, and FMU-EpCAM-4F6). Our previous work indicated that all of the antibodies could be used to stain colon carcinoma tissues for immunohistochemistry [20], suggesting that they have the potential to become anticancer drugs. In this work, we used 2A9 and 2D7 as candidate antibodies to prepare scFv. FCM analysis showed that 2A9 had relatively higher binding ability to EpCAM on the surface of two EpCAM-positive cells. Similar results were observed in SMMC-7721 cells (data not shown). So we selected 2A9 as the parental antibody to construct scFv2A9. The constructed expression vector pGEX4T1-scFv2A9-PE was finally identified by DNA sequencing.

After purification and identification, we performed flow cytometry analysis and ELISA to measure immunotoxin activity. The original pGEX-4T3-EpCAM and the immunotoxin all have a GST tag and can be hardly distinguished by anti-GST antibody. To detect its antigen-binding activity, we expressed the recombinant protein HIS-EpCAM as an antigen. ELISA and flow cytometry analysis confirmed functionality, in which the APE can detect the EpCAM molecule as 2A9, though with relatively lower activity. This was consistent with flow cytometry results that 2A9 and APE can bind the natural EpCAM with different binding abilities. Characterization of the specificity and binding affinity of APE was also carried out with a competitive binding assay with the monoclonal 2A9 antibody and APE. To confirm that APE recognizes the same extracellular EpCAM epitope as the 2A9 mAb, APE was used to block the binding of the 2A9 monoclonal antibody. The results (data not shown) confirmed that APE could compete with 2A9 to certain extent, but incompletely. Previous studies demonstrated that high affinity anti-EpCAM antibodies could cause toxic effects (phase I trials) [27, 28], while antibodies with moderate affinity to EpCAM-positive cancers efficiently mediated both antibody-dependent cellular cytotoxicity and complement-mediated cytotoxicity [29–31]. We must characterize the anticancer activity of the immunotoxin.

The immunotoxin can recognize EpCAM, and whether it is cytotoxic to EpCAM-positive tumor cells must be confirmed. MTT assay demonstrated that APE induced a 50% reduction of viability (IC_50_) of HHCC cells at a concentration of 50 pM, compared to control PE (>5000 pM). These compounds did not affect other cancer cells. Previous work suggested that immunotoxin cytotoxicity was related to its cellular internalization [32]. The immunotoxin has a C-terminal KDEL motif, which can localize it to the endoplasmic reticulum [33]. We observed this localization in HHCC cells by immunofluorescence and this suggested that the immunotoxin might internalize to cells and transport to the endoplasmic reticulum to exert its function. This finding was consistent with a study on KDEL receptor [34]. In addition, the internalization of the immunotoxin to Colo205 was also observed by immunofluorescence, and the immunotoxin was not detectable in cells after 24 h of incubation (data not shown).

The recombinant protein was expressed in* E. coli* cells as soluble form and as an inclusion body form (mainly). The soluble form of the protein maintained the natural structure and function and it could be used for function analysis [35]. We purified the soluble protein from 4 L bacteria and 2 mg of purified APE was collected, a yield somewhat lower than previous reports [36, 37]. Because the inclusion body was the main form of the immunotoxin, in follow-up work, we will purify the protein from inclusion bodies [38].

Immunotoxin, comprised of scFv and toxin, has many advantages compared with other therapeutics, such as small molecular weight, fewer side effects, a simple preparation method, and low production cost. If the scFv used to construct an immunotoxin can be fully humanized, the therapeutic potential of the immunotoxin will be powerful.

In conclusion, we have successfully developed an immunotoxin made of a single-chain variable fragment (scFv), derived from EpCAM monoclonal antibody FMU-EpCAM-2A9 and PE38KDEL. Its antigen-binding ability and cytotoxicity have been confirmed* in vitro*. Future work will include optimization of protein production, further development and testing of immunotoxin-based targeted therapies in animal models, and modification of the immunotoxin to decrease immunogenicity and toxicity.

## Figures and Tables

**Table 1:** Sequences of the primers used in this study are listed as A to L.

Primers	Sequence
A	tgaggagacggtgaccgtggtcccttggccccag
B	aggtsmarctgcagsagtcwgg
C	gttagatctccagcttggtccc
D	gacattcagctgacccagtctcca
E	gc**ggatcc**gaggtgaagctrcagcagt
F	cg**gtcgac**tgaggagacrgtgaccgtkg
G	cg**gtcgac**ggtggtggtggttctggtggtggtggttctggtggtggtggttctgatgttgtgctgacccagtctccactcactttgt
H	gc**aagctt**gatctccagcttggtccctcc
I	gc**gaattc**ggtggtggtggttctggtggtggtggttctggtggtggtggttctgcggccgcttctggaggt
J	cg**ctcgag**tcacagttcgtctttcggcggtttg
K	gc**ggtacc**caggaagaatgtgtctgtg
L	cg**aagctt**accagcttttagaccctg

B and F were degenerate primers; the degenerate base codes are as follows: s:c/g; m:a/c; r:a/g; w:a/t; k:g/t.
